# Augmented reality-based electrode guidance system for reliable electroencephalography

**DOI:** 10.1186/s12938-018-0500-x

**Published:** 2018-05-24

**Authors:** Chanho Song, Sangseo Jeon, Seongpung Lee, Ho-Gun Ha, Jonghyun Kim, Jaesung Hong

**Affiliations:** Department of Robotics Engineering, DGIST, Techno jungang-daero, Daegu, Republic of Korea

**Keywords:** Electroencephalography, Electrode positioning international 10–20 system, Augmented reality, Surface registration

## Abstract

**Background:**

In longitudinal electroencephalography (EEG) studies, repeatable electrode positioning is essential for reliable EEG assessment. Conventional methods use anatomical landmarks as fiducial locations for the electrode placement. Since the landmarks are manually identified, the EEG assessment is inevitably unreliable because of individual variations among the subjects and the examiners. To overcome this unreliability, an augmented reality (AR) visualization-based electrode guidance system was proposed.

**Methods:**

The proposed electrode guidance system is based on AR visualization to replace the manual electrode positioning. After scanning and registration of the facial surface of a subject by an RGB-D camera, the AR of the initial electrode positions as reference positions is overlapped with the current electrode positions in real time. Thus, it can guide the position of the subsequently placed electrodes with high repeatability.

**Results:**

The experimental results with the phantom show that the repeatability of the electrode positioning was improved compared to that of the conventional 10–20 positioning system.

**Conclusion:**

The proposed AR guidance system improves the electrode positioning performance with a cost-effective system, which uses only RGB-D camera. This system can be used as an alternative to the international 10–20 system.

## Background

Electroencephalography (EEG) is a neuroimaging technique that is frequently used to measure the neural activity in the brain. The standardized positioning of electrodes is essential in longitudinal EEG studies to minimize the test–retest and inter-examiner variability [1, 2] because even a small positioning error on the scalp can cause large changes in the measured electric potentials [1]. Thus, it is necessary to maintain consistent electrode locations over long-term trials to facilitate reliable EEG assessments.

Several studies have been proposed to reproducibly position EEG electrodes. The international 10–20 system is the de-facto standard electrode-positioning method, which relies on the manual identification of four anatomical landmarks [3]. Several approaches were proposed using the anatomical landmarks of the international 10–20 system. Echallier and Perrin [4] proposed a computer-assisted electrode-positioning system. An ultrasonic digitizer was used to define a reference coordinate system based on the aforementioned four anatomical landmarks. Giacometti et al. [2] developed a cap for the standard electrode positioning, which enabled the measurement of both EEG and functional near-infrared spectroscopy (fNIRS). The cap was placed using the 10–10 system, which is an expanded version of the international 10–20 system. Tsuzuki et al. [5] proposed the MinR 10–20 system that used landmarks of nasion, right and left preauricular points and posterior point on the occipital protuberance. Xiao et al. [6] proposed a semi-automatic 10–20 identification method using the virtual 10–20 landmark determination in the computational space of reconstructed head surface. The virtual landmarks were identified using a visually guided navigation system, which used a magnetic digitizer.

These systems require manual identification of anatomical landmarks prior to the electrode positioning. Thus, they potentially include non-negligible human error because of the structural ambiguity of anatomical landmarks [7, 8].

To address this issue, Jeon and Chien [9] proposed a preliminary study for precise image-guided electrode placement. A vision-based position tracker and a laser scanner were used for electrode guidance. Based on the serial coordinate registration, without the manual 10–20 landmarks identification, precise electrode repositioning was demonstrated. In this study, a simple electrode-positioning system was proposed using an augmented reality (AR) visualization technique. Since the proposed method can support real-time registration using the face surface, it did not require the fiducials for the registration or a reference marker attached to the subject’s body.

A phantom study was conducted to evaluate the effectiveness of the proposed system compared with that of the international 10–20 system. Although several studies have examined AR visualization [10–12], to the best of the authors knowledge, this is the first EEG study to utilize an AR visualization technique for precise electrode positioning.

## Methods

The international 10–20 system is a standard EEG electrode-positioning method, which is generally adopted in related fields [3, 7, 13–15]. Four landmarks are manually identified by clinicians for electrode positioning with the international 10–20 system: nasion, inion, and left/right preauricular points. The midline that connects the nasion to the inion and the central line that connects the left preauricular point to the right preauricular point are subsequently measured. Anterior–posterior planes and central coronal planes based on the two reference lines are determined. The electrodes are placed on lattice points, which are defined as intersections of the planes on the scalp at 10 and 20% intervals [3].

However, an unreliable EEG assessment can be generated by individual variations in positioning electrodes. To solve this problem, an AR-based electrode guidance system was developed. AR is a technique to superimpose a virtual object onto a real object [16]. In medical imaging, AR is used to visualize the medical information superimposed on the patients image. For example, in surgical navigation, the patients risk factors and target organ can be provided to the surgeon via AR during the surgery [17–19]. In this study, we visualize the electrode location using AR and improve the repeatability of the EEG electrode placement in the long-term EEG study.

Several techniques are essential for the AR environment, including marker tracking, tracker-camera calibration, and patient-to-image registration [20, 21]. In this study, an RGB-D camera (Realsense f200, Intel, California, USA) is used as the tracker. 3D point data of the subjects surface including a head and a face is acquired using the RGB-D camera and used for the surface registration. Since the RGB-D camera coordination is defined with respect to the subjects facial surface, the transformation between the current RGB-D camera coordination and the pre-acquired RGB-D camera coordination is calculated through the surface registration. Therefore, without using an optical tracking system or external marker, the positions of the initially placed electrodes (pre-acquired RGB-D image) are superimposed on the patients head (current RGB-D image) with respect to the subjects facial surface.

### System overview

The proposed system uses an RGB-D camera to scan the electrodes and anatomical features. Specifically, a Realsense camera software development kit was used to interface the RGB-D camera and acquire the scanned 3D points. A visualization toolkit and a point cloud library were used to visualize the processed data and handle the 3D points, respectively [22]. The software was executed in a workstation equipped with an Intel Core i7 CPU, 32 GB RAM, and NVIDIA GeForce GTX 970 GPU.

#### System workflow

**Fig. 1:**
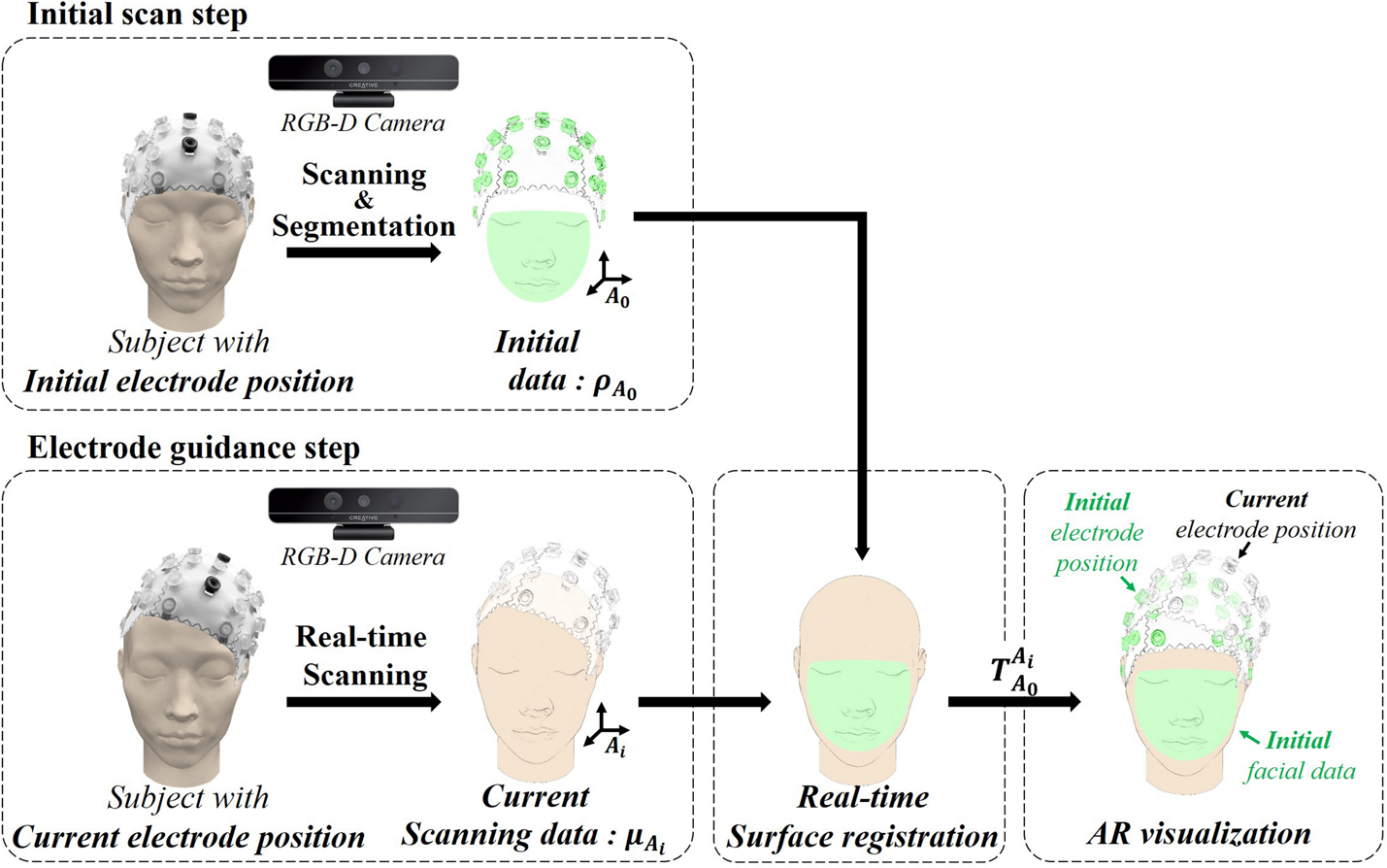
Workflow of the proposed electrode guidance system. In the initial scan step, $A_{0}$ is the coordinate system of initial scan data, $A_{i}$ is the current scanning coordinate system with an index of surface registration, *i*. By real-time surface registration, the transformation between the initial coordinate system and the current coordinate system is updated

The workflow of the proposed system for electrode guidance is shown in Fig. 1, which consists of an initial scan and electrode guidance steps.

#### Initial scan step

An electrode cap is initially positioned on the subjects head using the international 10–20 system. The head, including the facial surface and electrodes, is scanned using the RGB-D camera. Next, the facial surface and electrodes are separately segmented using an open-source software program (CloudCompare, France). The facial surface is used for surface registration, and the electrodes are used to set the initial electrode locations in the electrode guidance setup, which is visualized using AR.

#### Electrode guidance step

The subsequently placed electrodes are repositioned according to the AR guidance without using the international 10–20 system. To simultaneously track the facial surface of the subject and implement AR visualization, the scanning and registration should be processed in real time. More details on surface registration are described in the real-time surface registration section. The AR visualization simultaneously superimposes the initial electrode position over the current position current electrode position. Thus, the clinician can place the current electrodes at the position of the initially placed electrodes with high repeatability.

### Real-time surface registration

To implement AR visualization with respect to the facial surface of the subject, the surface registration between the initial facial surface and the current scanning data is repeatedly performed. The surface registration is performed by using the iterative closest point (ICP) algorithm. The ICP algorithm solves for a transformation from the target to source coordinate systems using the surface data [23, 24]. In the proposed system, the initial facial surface, which is acquired from the initial scan step, is used as the source data, and the current scanning data are used as the target data. Although the initial and current scan data from the RGB-D camera are actually defined on the same coordinates, which are the image coordinates, they are separated into two different sets of coordinates: initial scan data and current scanning data coordinates. During the surface registration, the transformation between the coordinates of the initial scan data and current scanning data is continuously calculated, as shown in Fig. 1. For the real-time surface registration, the transformation of the prior registration result is used to update the initial facial surface, and the surface registration is processed between the updated initial facial surface and the current facial surface. The ICP-based surface registration is used to update the transformation, $T_{A_{{i}\,-\,{1}}}^{A_{i}},$ which denotes a relationship between the current scan coordinate system and the initial scan coordinate system.

1 $$\begin{aligned} T_{A_{0}}^{A_{i}} = T_{A_{0}}^{A_{{i}\,-\,{1}}}\, T_{A_{{i}\,-\,{1}}}^{A_{i}}, \end{aligned}$$

where ${A_{0}}$ is the coordinate system of the initial scan data, ${A_{i}}$ is the current scanning coordinate system, and $A_{{i}\,-\,{1}}$ is the previously updated initial coordinate system. To avoid the local minima problem and reduce the time taken for the ICP-based surface registration, $T_{A_{0}}^{A_{{i}\,-\,{1}}}$ is used for the initial alignment. 

**Fig. 2:**
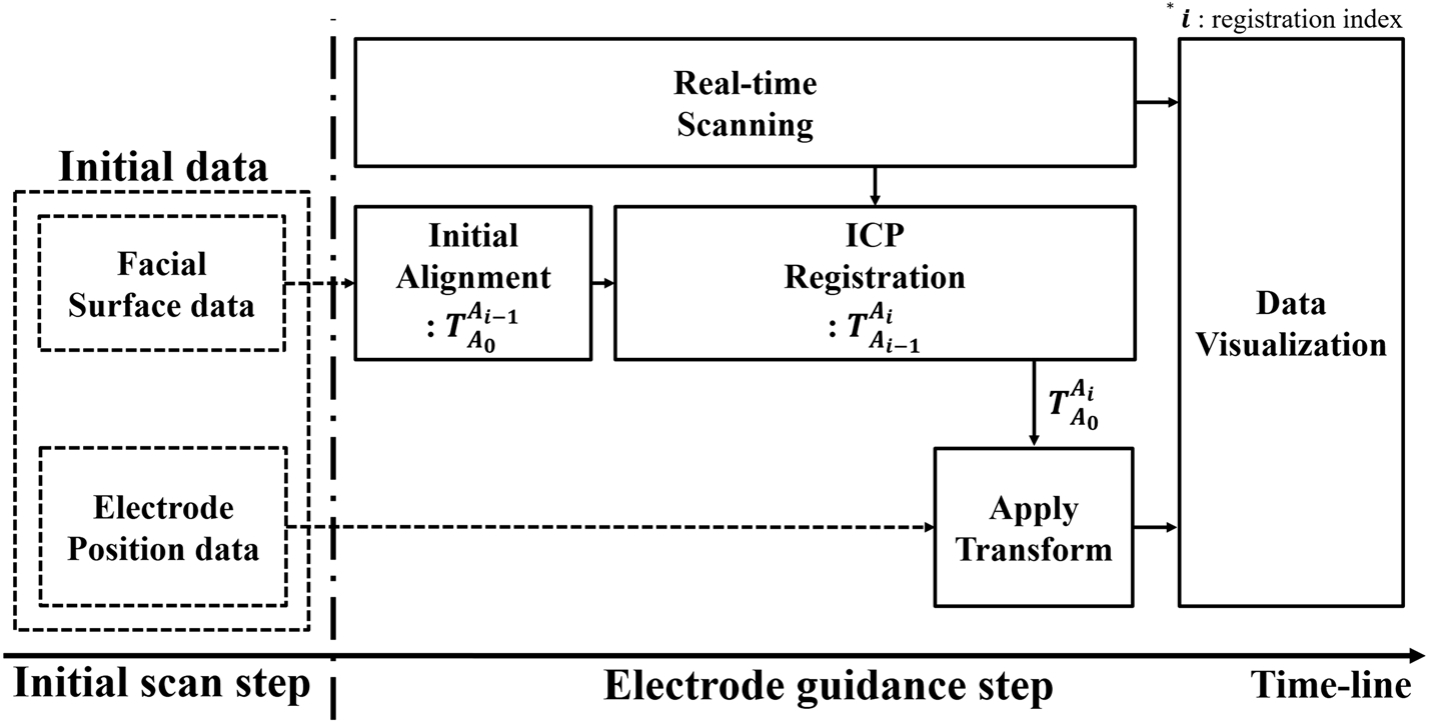
Pipeline of real-time surface registration

The final transformation is applied; thus, the initial scan with respect to ${A_{0}}$ is transformed to ${A_{i}}$, as shown in Eq. (2):

2 $$\begin{aligned} {}_{}^{A_{i}}{\rho } = T_{A_{0}}^{A_{i}}\, {}_{}^{A_{0}}{\rho }, \end{aligned}$$

where ${}_{}^{A_{i}}{\rho }$ and ${}_{}^{A_{0}}{\rho }$ denote the updated initial electrode position data and initial electrode position data, respectively. During the surface registration, an ICP algorithm is continuously performed with more than 10,000 corresponding points, so a heavy computational cost entails. When scanning and surface registration are serially processed, the updating rate of the display is significantly reduced. Thus, we executed scanning and surface registration in parallel, as shown in Fig. 2, to increase the updating rate.

### Experimental setup

**Fig. 3:**
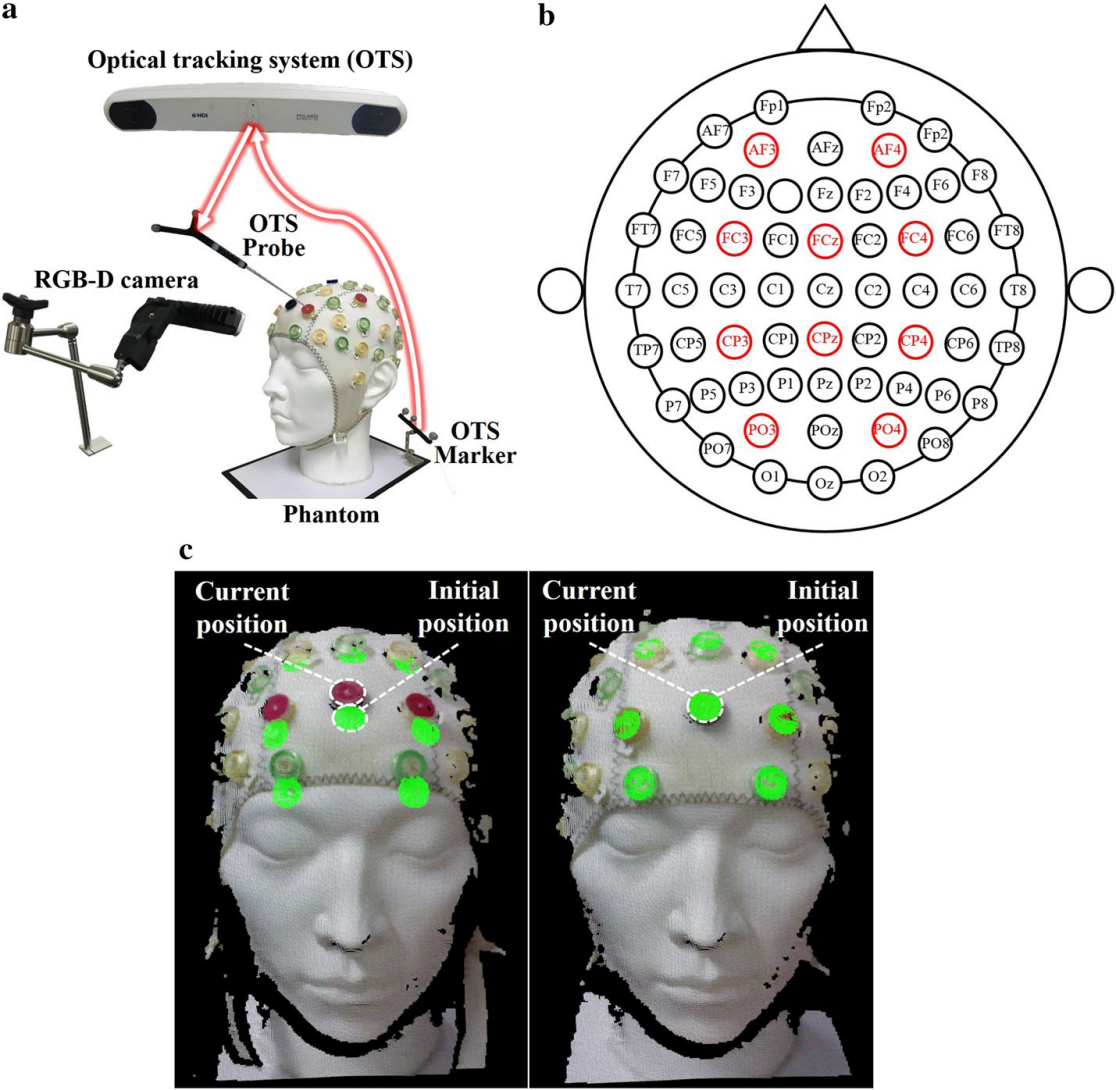
**a** Experimental setup for the electrode positioning evaluation. **b** Labelled red target electrodes on an EEG cap. **c** Electrode guidance display (left) during and (right) after the positioning

Electrode positioning experiments were performed to verify the repeatability of the proposed electrode guidance system. The experimental setup to measure the electrode positioning error is shown in Fig. 3a. A commercial electrode cap with 64 channels (actiCAP, Easycap, Herrsching, Germany) was used. A head phantom including four anatomical landmarks was used as a subject for repeated trials (60 times).

Ten target electrodes were labelled on the EEG cap to measure the positioning error: AF3, AF4, FC3, FCz, FC4, CP3, CPz, Cp4, PO3, and PO4, as shown in Fig 3b. To measure the 3D coordinates of the electrode position, an optical tracking system (OTS) (Polaris Vicra, Northern Digital Inc., Waterloo, Canada) was used with a high tracking accuracy (root mean square 0.35 mm). An OTS marker was attached to the phantom to track the head phantom. The electrode positions with respect to the OTS marker on the phantom were acquired using a hand-held OTS probe. The OTS marker on the phantom was fixed and used as a reference coordinate system during the experiment. Three participants were involved in the experiment to place the electrode cap on the phantom. The initial positioning of electrodes was performed using the international 10–20 system, and the initial positions of the electrodes were used as the gold standard to calculate the positioning error. Then, each participant repositions the electrode cap 10 times using the proposed system and international 10–20 system. When the proposed system is used, an AR image of the initial electrode positions is shown, as shown in Fig. 3c. The positioning error is calculated for the 10 pre-defined electrode locations as follows:

3 $$\begin{aligned} Positioning\;error = \left| \left| {p}-{p'}\right| \right| \end{aligned}$$

where ||.|| denotes an absolute value, *p* denotes the coordinates of the measured target points from the electrode positioning methods such as the proposed method and conventional method, and $p'$ is the gold standard electrode positions.

## Results

### Electrode positioning error

**Fig. 4:**
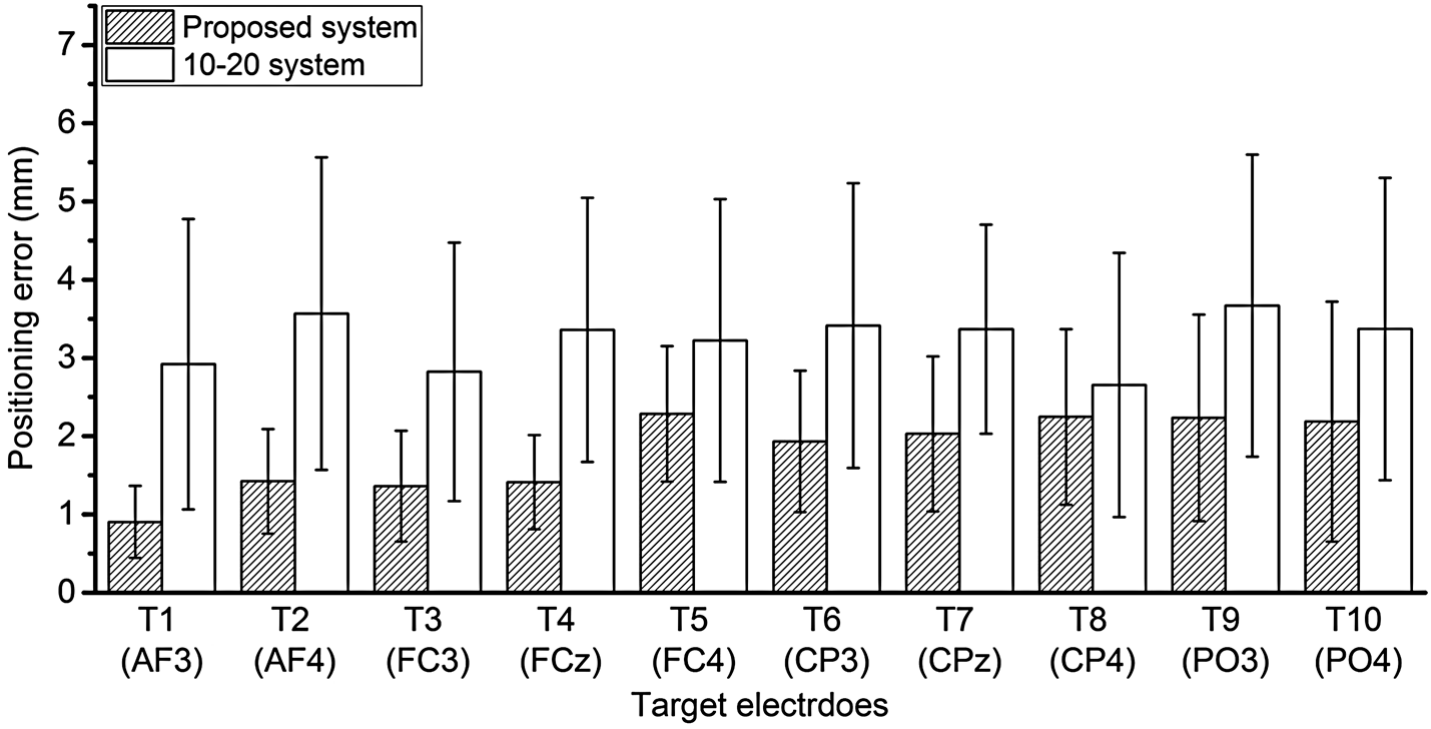
Comparison of positioning error at each target electrode between the proposed system and the 10–20 system

In the phantom study, the positioning error of the proposed system was compared with that of the international 10–20 system. The mean positioning error was 1.8 ± 1.06 mm for the proposed system and 3.24 ± 1.78 mm for the international 10–20 system. Figure 4 shows a comparison of the positioning errors at each target electrode. Both mean and standard deviation of all target positioning errors from the proposed system were smaller than those of the conventional system. In particular, the errors measured at the electrodes located on the frontal scalp, i.e., AF3, AF4, FC3, and FCz, were smaller than those measured at the relatively posterior electrodes. The results indicate that the repeatability of electrode positioning using the international 10–20 system is unreliable because of the individual variations of each clinician to locate electrodes, whereas the repeatability is improved in the proposed system.

**Fig. 5:**
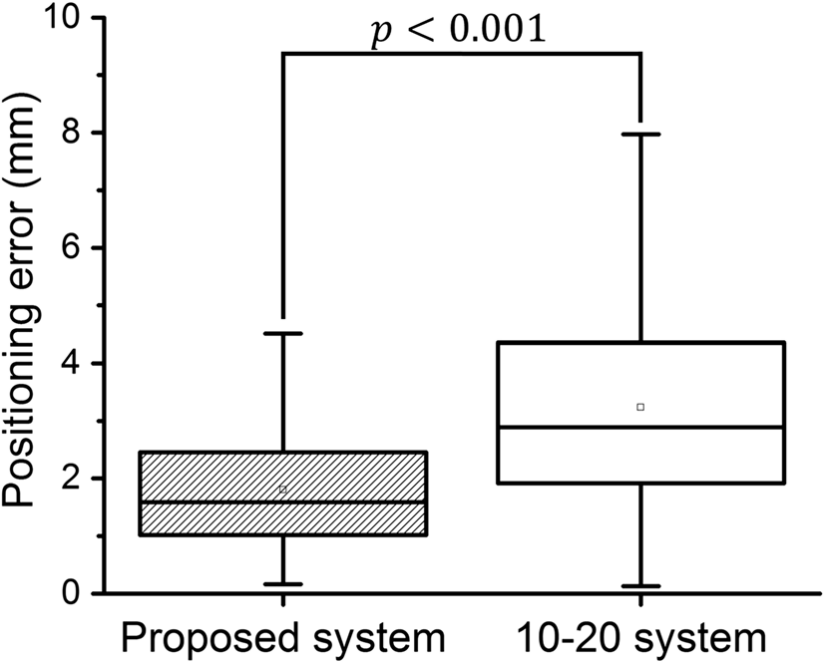
Comparison of overall positioning error between the proposed system and the 10–20 system

A statistical analysis was performed using the OriginLab software (OriginPro 2015, Northampton, Massachusetts, USA). Kolmogorov–Smirnov normality test at the 0.001 significance level demonstrates that both experimental results were drawn from a normally distributed population. The independent t-test indicates that the positioning error of the proposed system is significantly different from that of the international 10–20 system (*p* < 0.001), as shown in Fig. 5.

### Real-time registration performance

The registration error and computational time were measured over 50 times to evaluate the performance of the real-time surface registration throughout the experiments. The mean registration error was 0.37 mm, and the mean registration time was 0.16 s, which is equivalent to 6.25 frames/s with respect to the update rate of the display.

## Discussion

In the proposed system, the surface registration is performed for AR visualization. The parallel pipeline enables one to visualize the scanning data that represent the initial electrode position in real time despite the high computational cost of the ICP. However, the ICP surface registration was performed with a single parameter condition throughout the experiments, which included the point-to-point error metric and uniform data sampling. Therefore, more investigations on the registration accuracy and resultant positioning precision with different parameter conditions are required.

Considering the system configuration, the conventional 10–20 positioning systems is simple and inexpensive, but an unreliable electrode positioning can occur from the manual identification of the anatomical landmarks [7, 8, 13–15]. To address this issue, an extra device such as a vision-based position tracker, a commercial ultrasonic digitizer or a magnetic digitizer has been used so far [4, 6, 9]. Compared to those system, only an RGB-D camera is necessary in the proposed system. An RGB-D camera is more cost-effective than a tracker or digitizer, and additional accessories are not required.

To verify the proposed system, three participants performed the experiments in 30 trials. Although the number of participants is small, our main concern is to address the inter-session variation issue. In a longitudinal EEG study, the manual identification of the anatomical landmarks can cause inter-session variations. The experiment focused on verifying the improvement in repeatability of the electrode positioning at each participant.

In the phantom study, the proposed system exhibited a smaller positioning error than the conventional system. The improved positioning precision is attributed to the exclusion of human error and the use of morphologically invariant anatomical surface information scanned by the RGB-D camera. In other words, the proposed system reduces the ambiguity of anatomical landmarks for electrode positioning. Conversely, the positioning errors measured in the frontal-scalp-located electrodes are smaller than those of the posterior-scalp-located electrodes. This tendency can result from the narrow field of view (FOV) of the RGB-D camera. The narrow FOV enables the proposed system to only guide the electrodes within the FOV.

The electrodes on the elastic EEG cap, which were used in the study, are also not rigidly fixed with respect to one another. Unexpected changes in their relative positions during the guidance can yield inaccurate positioning with respect to the electrodes that are not directly guided by the AR system. If a non-elastic EEG cap is used in the proposed system, the accuracy can be improved. The use of a stereo or multi-RGB-D camera system can also improve the accuracy of the proposed system.

## Conclusion

In this study, an electrode guidance system with high repeatability of electrode positioning was proposed based on the AR visualization. The experimental results indicate that the proposed system outperforms the conventional methods in terms of repeatability. We also improved the performance with a cost-effective system by using only an RGB-D camera. Although the performance of the proposed system is limited by the narrow FOV and relatively low image resolution of the adopted RGB-D camera, this problem can be solved by using a high-resolution stereo camera system. We expect that the concept of the proposed system will be applied for standard EEG studies and similar applications such as a near-infrared spectroscopy measurement.

## Abbreviations

EEG: electroencephalography
fNIRS: functional near-infrared spectroscopy
AR: augmented reality
ICP: iterative closest point
OTS: optical tracking system
FOV: field of view
